# Medulloblastoma in adults – reviewing the literature from a surgeon’s point of view

**DOI:** 10.18632/aging.202568

**Published:** 2021-01-26

**Authors:** Thomas Eibl, Alexander Hammer, Eduard Yakubov, Cristiane Blechschmidt, Alexander Kalisch, Hans-Herbert Steiner

**Affiliations:** 1 Department of Neurosurgery, Paracelsus Medical University, Nuremberg 90471, Bavaria, Germany; 2 Department of Neuropathology, Paracelsus Medical University, Nuremberg 90471, Bavaria, Germany; 3 Department of Oncology, Paracelsus Medical University, Nuremberg 90471, Bavaria, Germany

**Keywords:** adult medulloblastoma, medulloblastoma, posterior fosa tumor, cerebellar tumor, aging

## Abstract

Medulloblastoma is a common primary brain tumor in children but it is a rare cancer in adult patients. We reviewed the literature, searching PubMed for articles on this rare tumor entity, with a focus on tumor biology, advanced neurosurgical opportunities for safe tumor resection, and multimodal treatment options. Adult medulloblastoma occurs at a rate of 0.6 per one million people per year. There is a slight disparity between male and female patients, and patients with a fair skin tone are more likely to have a medulloblastoma. Patients present with cerebellar signs and signs of elevated intracranial pressure. Diagnostic efforts should consist of cerebral MRI and MRI of the spinal axis. Cerebrospinal fluid should be investigated to look for tumor dissemination. Medulloblastoma tumors can be classified as classic, desmoplastic, anaplastic, and large cell, according to the WHO tumor classification. Molecular subgroups include WNT, SHH, group 3, and group 4 tumors. Further molecular analyses suggest that there are several subgroups within the four existing subgroups, with significant differences in patient age, frequency of metastatic spread, and patient survival. As molecular markers have started to play an increasing role in determining treatment strategies and prognosis, their importance has increased rapidly. Treatment options include microsurgical tumor resection and radiotherapy and, in addition, chemotherapy that respects the tumor biology of individual patients offers targeted therapeutic approaches. For neurosurgeons, intraoperative imaging and tumor fluorescence may improve resection rates. Disseminated disease, residual tumor after surgery, lower radiation dose, and low Karnofsky performance status are all suggestive of a poor outcome. Extraneural spread occurs only in very few cases. The reported 5-year-survival rates range between 60% and 80% for all adult medulloblastoma patients.

## INTRODUCTION

Medulloblastoma is a malignant primary brain tumor usually located in the posterior cerebral fossa. Most patients with medulloblastoma are children, but adolescents and adults may also present with this cerebellar tumor. Since adult medulloblastoma is an extremely rare disease, most investigations concern patients of younger age, especially investigations of molecular features and treatment protocols. Medulloblastoma is a well-known tumor in children and a lot of research has been carried out in order to understand the molecular biology and molecular subgroups, as well as the signal pathways, underlying this tumor. All this knowledge has led to treatment protocols for children and possibilities for targeted therapy approaches for selected patients. Due to the limited number of adult patients with medulloblastoma, data and randomized trials are lacking, and these patients are sometimes treated within pediatric trials. On the other hand, the published data suggest important differences between childhood and adult medulloblastoma, in terms of tumor biology, treatment strategies, and outcome predictors.

The incidence of medulloblastoma among all age groups is estimated at about 1.5 per million per year and the incidence of all primitive embryonal tumors is estimated to be about 2 per million per year [1, 2]. The incidence in adults is about 0.6 per million per year [2]. 1.5% of posterior fossa craniotomies among adult patients are for medulloblastoma [3], and the dispersion between males and females is about 1.5/1 for all embryonal tumors [1]. There is a slight disparity in the incidence of medulloblastoma between patients with different cultural backgrounds according to the SEER-register, and adults with a fair skin tone are more likely to have a medulloblastoma [2]. Most adult patients with medulloblastoma seem to be diagnosed between 20 and 40 years of age [4].

The aim of this review is to provide a short overview of this rare cancer, focusing on imaging features, opportunities for surgical treatment, molecular pathology, adjuvant treatment, and prognostic factors. Whenever possible, we have tried to enhance the differences between adult and childhood medulloblastoma, especially regarding prognostic factors and implications for therapeutic considerations. A summarized overview over this disease is provided in Table 1.

**Table 1:** Overview over the most relevant facts about adult medulloblastoma.

**Medulloblastoma in adults - quick overview**	
**Epidemiology**	
Incidence	0.6/1 000 000
Age peak	20 - 40 yrs
Male/Female	1.5/1
**Staging**	
	cranial MRI
	spinal MRI
	CSF cytology
**Treatment**	
Surgery	aim to achieve no residual tumor
Craniospinal Irradiation	up to 36 Gy
Posterior fossa boost	up to 55 Gy
Adjuvant Chemotherapy	different protocols
**Surveillance**	
	cranial MRI
	spinal MRI
	CSF cytology
**Prognostic factors (for worse outcome)**	
	male patients
	residual tumor after surgery
	metastatic spread
	lower radiation dose
	no chemotherapy
**Overall Survival**	
	60 - 80%/5 yrs
	55%/10 yrs

## Methods

In 2020, we reviewed the literature using a selective PubMed search on adult medulloblastoma with an emphasis on the factors mentioned above. We used the search terms “medulloblastoma in adults”, “adult medulloblastoma”, “medulloblastoma 5-ALA”, “vismodegib”, “vismodegib medulloblastoma” “medulloblastoma temozolomide”, “temozolomide”, “cerebellar tumor”, “medulloblastoma metastases”, “medulloblastoma complications”, “medulloblastoma desmoplastic”, “medulloblastoma hydrocephalus”, “posterior fossa tumor surgery”, “neuromonitoring posterior fossa”. We also searched cited references among the results and we also reviewed articles suggested by PubMed as similar articles among the search results. Articles were selected by title and/or abstract, published in English, availability of the full text, published date (preferred not published before 2000), and number of patients in clinical trials. Selected case reports were also included.

## Clinical presentation

The length of disease history of medulloblastoma patients is usually only up to a few weeks from the onset of symptoms up to admission to hospital [5–7]. Many patients with medulloblastoma are admitted to hospital with signs of intracranial pressure, including headache, nausea or disturbances of consciousness caused by hydrocephalus due to a tumor in the posterior fossa [6, 8]. Furthermore, a cerebellar mass lesion can cause vertigo, coordination disorders, ataxia or palsies of the cranial nerves, resulting in visual abnormities, oculomotor disorders and nystagmus [7, 9].

## Diagnostic and staging

As a first diagnostic procedure, a cranial CT-Scan is often performed. In the case of medulloblastoma patients, the tumor mass typically appears hyperdense on the CT scan [10, 11]. Further diagnostic exams should be carried out after the initial proof of a cerebellar tumorous mass. These include cerebral MRI and spinal MRI, as medulloblastomas tend to seed metastases along the spinal axis. Cerebrospinal fluid cytology should be carried out in order to detect leptomeningeal spread of the tumor. This is usually performed after debulking surgery to avoid cerebellar herniation.

Medulloblastoma in children arises from the vermis and is often located in the cerebellar midline [6]. In contrast to this tumor location, adult medulloblastoma can also be found in the cerebellar hemispheres [12].

In MR-Imaging, medulloblastomas usually present as enhancing tumors with sharp margins, sometimes with diffuse enhancement uptake [13, 14]. In T1-weighted sequences, medulloblastoma is typical iso- or hypointense compared to cerebellar tissue [14]. In T2-weighted images (T2WI), medulloblastomas are present as a heterogenous signal. In one series, classic and anaplastic medulloblastomas appeared hyperintense in T2WI [14]. Desmoplastic and medulloblastoma with extensive nodularity appeared isointense in T2WI [14]. In Diffusion Weighted Imaging (DWI), medulloblastomas suggest a restriction of diffusion [14], and DWI might be helpful to rule out other tumor entities as a differential diagnosis [13]. Cystic tumor components can be found in all medulloblastoma subtypes, whereas calcifications and hemorrhage are less common features [14]. MRI-findings derived from patients enrolled into the NOA-07-trial [15] revealed hemorrhage, hydrocephalus, and intraventricular metastases as sensitive parameters to identify WNT-activated medulloblastomas in adults [16].

Chang et al. developed a staging system for medulloblastoma based on the local tumor extent and metastases ([17] citation in [18]). This historic staging system is derived from the TNM-System for other tumor entities, where the T-stage describes local tumor infiltration (T1-T4) and the M-stage describes the extent of metastatic seeding, cerebrospinal fluid invasion, and distant metastases (M0-M4).

In adult patients there might be a prognostic relevance depending on the T-stage [19].

The Chang-Classification is nowadays of historic value and Medulloblastomas are classified according to the WHO and according to molecular features that will be discussed in the following.

Differential diagnoses for medulloblastoma include cerebellar tumorous lesions, such as ependymoma, hemangioblastoma, vestibular schwannoma, glioblastoma, choroid plexus papilloma, cerebellar lymphoma and cerebellar metastases of systemic tumors [8].

## Histopathology

Medulloblastomas are WHO grade IV tumors [20, 21]. According to the WHO classification, medulloblastoma can be divided into different histopathological subgroups [20, 21]. These are described as classic, desmoplastic, anaplastic, and large cell medulloblastoma, as well as medulloblastoma with extensive nodularity. In recent years, the importance of molecular subgroups has increased in tumor treatment due to possible targeted therapies and the prognostic influence of certain molecular subtypes. In medulloblastoma in general, four molecular variants are of great importance: WNT, SHH, group 3, and group 4 [22].

The 2016 WHO classification gives an overview of the histological and genetical variants and a pathologist should make a diagnosis respecting both molecular aspects and the phenotype of the tumor. The classification includes WNT, SHH, and TP53 as molecular markers [21]. The WHO classification shows most integrated medulloblastoma diagnoses. The classic and anaplastic/large-cell histologic subtypes can be found with any molecular constellation, whereas desmoplastic medulloblastomas are usually SHH-activated [23]. On the other hand, WNT positive tumors usually show a classic histology [24].

Histopathological tumor diagnosis and classification is the same in children and in adults, but the distribution of tumor subtypes is different in adult medulloblastoma patients [22, 23]. Children are most often diagnosed with the classic medulloblastoma subtype [23]. Infants and adults are more often diagnosed with a desmoplastic subtype [23]. Authors found three subtypes of adult medulloblastoma, WNT, SHH and Group D/4, whereas Group 3 medulloblastomas are extremely rare in adult patients [25, 26]. On the other hand, desmoplastic and SHH-activated medulloblastomas are the most common subtype in adults. Another approach suggests more molecular subtypes exist within the four molecular groups [27]. This more detailed molecular classification subclassifies each subtype into another two, three, or four subtypes, respecting other molecular markers such as MYC, MYCN, and CDK6. This investigation also indicated an age-related distribution within the subgroups, and the detailed molecular subtypes were also found to influence metastases and overall prognosis. WNT beta and SHH delta subtypes seem to be exclusively found in adult patients [27].

Northcott et al. [28] demonstrated that further molecular and cytogenetic differences exist in the pediatric and adult cohort for Sonic Hedgehog-activated medulloblastomas. For instance, chromosome 10q deletion and MYCN amplification occurred more often in pediatric than in adult SHH-activated medulloblastomas [28]. Regarding prognostic factors, metastatic spread was found to be of prognostic significance in adults but not in pediatric patients with SHH-activated medulloblastoma [28]. Korshunov et al. [12] also found several molecular differences between adult and childhood medulloblastoma. They showed evidence for CDK6, 10q loss, and 17q gain as prognostic markers in adults and could not find a prognostic significance for MYC/MYCN amplification [12, 29]. Among WNT and SHH-activated tumors, tumors with TP53 mutation are peculiar, since these tumors occur in a different age group (mostly between 5-18 years), show a higher rate of anaplasia and indicate an inferior prognosis for the patients in the SHH subgroup [30]. Further research revealed several additional molecular markers for WNT-and SHH-activated medulloblastomas [24]. WNT-positive medulloblastomas were strongly linked to nuclear and cytoplasmatic immunoreactivity for b-catenin, filamin A, and YAP1, whereas SHH-activated tumors showed immunoreactivity for GAB1, YAP1, and filamin A [24]. Non-WNT/-SHH tumors were not positive for GAB1 and YAP1-markers and could, therefore, be distinguished from the other subgroups [24].

Research found evidence for major differences in adult medulloblastomas compared to the pediatric tumor concerning distribution of subtypes, genetic alterations and prognostic factors which should be taken into account for making a histological diagnosis and as prognostic markers as shown above.

Given that there are at least four histological and at least four molecular subgroups for this tumor entity, the question arises if all medulloblastomas have the same origin of tumor growth. WNT and SHH-tumors seem to arise from different precursor cells, either in the dorsal brainstem, the fourth ventricle, or the cerebellum [31].

Trials investigating imaging criteria and histological subtype have revealed a coherence between specific imaging features, such as tumor location, imaging signal intensity, and molecular subtype in children [14, 32, 33]. WNT medulloblastomas were found in the cerebellopontine angle cistern and cerebellar peduncle, SHH-activated medulloblastomas were located within the cerebellar hemispheres, and group 3 and group 4 tumors were located in the midline and fourth ventricle [33, 34]. Perreault et al. showed that group 4 medulloblastomas are non-enhancing in about half of the patients, with a high predictive value for group 4 tumors [34]. In children, WNT medulloblastomas can also be found at Foramen Luschka and the fourth ventricle [32, 35]. Typical findings for intracerebral tumors such as edema, necrosis or cystic formations are mostly non subgroup specific [34].

## Treatment

Therapeutic strategies include tumor surgery, radiation, and chemotherapy [19]. Treatment strategies should be adapted to the patient’s clinical status and tumor biology.

## Surgery

Perioperatively, patients should be monitored on an intensive care unit as posterior fossa tumors can cause acute hydrocephalus and tonsillar herniation [36]. In cases of patients presenting with hydrocephalus and clinical signs such as depression of consciousness, it is sensible to place an extraventricular drain into a lateral ventricle in order to carefully relieve intracranial pressure.

Tumor resection is performed in order to gain tumor tissue for histopathological examination, molecular classification, and diagnosis. The prognostic value of total tumor resection is not definitely clear in adult patients. However, guidelines recommend a gross tumor resection if possible [5, 19, 37] and Call et al. could find a prognostic benefit for patients who undergo gross tumor removal [38]. Data show a correlation between residual tumor and T-stage, suggesting that a total and radical resection of greater tumor mass in the posterior cranial fossa is often not possible as neurosurgeons try to keep patient safety and neurological outcomes at an acceptable level. Leaving no residual tumor should be achieved if the risk for complications is acceptable and the functional outcome for the patient is favorable [19]. Total tumor resection also helps to reduce intracranial pressure, pressure to the brainstem, and hydrocephalus. Therefore, gross tumor resection treats the symptoms caused by the tumor.

Surgery for medulloblastoma is usually performed via a midline infratentorial craniotomy in a sitting or lateral-oblique (park-bench)-position using a neuronavigation system [36, 39]. Positioning of the patient does not influence surgical success in cerebellar tumor resection [40]. Positioning-related complications, such as air-embolism in the sitting position, need to be respected [39, 41, 42]. There seem to be no special patient-dependent risk factors for air embolism and the semi-sitting patient positioning appears not to influence complication rates of posterior fossa surgery [39]. Other analyses even show less surgical complications, such as bleeding, aside from air embolism for the semi-sitting position [41, 43].

Intraoperative monitoring may be applied for infratentorial lesions, offering good negative predictive values for postoperative deficits [44, 45]. Monitoring for infratentorial microsurgery usually includes somatosensory evoked potentials and motoric evoked potentials (SEP and MEP) [45]. Slotty et al. [44] presented a series of 305 patients undergoing posterior fossa craniotomy for several pathologies, and intraoperative monitoring of cranial nerves and SEP/MEP were used [44]. The authors found evidence for intraoperative signal alterations for lesions in distinct locations such as brainstem, and midline lesions, whereas tumor surgery within cerebellar hemispheres appeared to be safer concerning postoperative neurologic sequelae [44]. In the future, further developments in neuromonitoring and tools for dynamic mapping will probably increase safe resections with reduced rates of patients harboring a postoperative deficit [46].

The complications of midline suboccipital craniotomy and tumor resection include bleeding, hydrocephalus, palsies of lower cranial nerves, ataxia, visual disturbances, and dysphagia [47].

Intraoperative tumor fluorescence (5-aminolevulinic-Acid) is an established method in glioma surgery and intraoperative fluorescence angiography (indocyanine-green) is in routine use for neurovascular procedures. However, neither is widely used for the resection of non-glial tumors [48–51]. In glioblastoma removal, 5-aminolevulinic-acid guided surgery has become a standard procedure improving rates of tumor removal [52–54]. There have been published case series and case reports trying to adapt intraoperative fluorescence in medulloblastoma surgery as well but there is no routine use of intraoperative fluorescence. *In vitro* studies suggest a possible fluorescence of medulloblastoma cell lines which is weaker than in glioblastoma cells [55]. Published *in vivo* trials suggest that the fluorescence caused by medulloblastoma cells is too inconsistent and only 20-25% of the tumors in reported case series show adequate fluorescence to help neurosurgeons perform a better tumor resection [56, 57].

Intraoperative MRI is also commonly used for resection of glial tumors, if available. With the use of intraoperative MRI, total tumor resection rates of glial tumors are significantly higher [48]. Data has also demonstrated the advantages of intraoperative MRI for pediatric tumors and posterior fossa tumors [58, 59]. However, data concerning intraoperative MRI, especially for the resection of medulloblastomas, is lacking but it seems sensible to perform intraoperative imaging for increased surgical quality and patient safety during tumor resection. On the other hand, intraoperative imaging requires a high amount of expertise, lengthens the time of anesthesia, and increases the time the patient spends in the operating theatre.

Some patients will need cerebrospinal fluid diversion as the cerebellar tumor causes hydrocephalus. It may be sensible to perform a third ventriculostomy if possible [60]. If patients need a ventriculoperitoneal shunt, there will be an increased risk for peritoneal metastases due to tumor cells seeding via cerebrospinal fluid diversion [61–63]. In a pediatric cohort, around 20% of the patients required cerebrospinal fluid diversion [64]. For adult patients with any posterior fossa lesions, preoperative presence of hydrocephalus is a risk-factor for shunt-dependency after tumor resection [3].

A postoperative cranial MRI should be performed within 48 hours after surgery documenting the extend of resection and revealing bleeding or other complications [19]. In some cases, a second look surgery might be considered if there is more than 1.5cm^2^ of residual tumor after first surgery [19]. Investigations among childhood medulloblastoma found a negative prognostic value for a residual tumor greater than 1.5cm^2^ on postoperative imaging [37]. When studying the molecular subgroups of medulloblastoma, gross or near total resection was only associated with a better outcome for group 4 patients [37].

In adult patients the extent of resection significantly influences the overall survival of the patients [12].

In cases of repeated microsurgery for posterior fossa tumors, no statistically increased risk of morbidity or mortality due to a second craniotomy was found [65].

## Radiotherapy

Surgical removal of the tumor should be followed by craniospinal irradiation and a boost to the posterior fossa in any patient. Radiotherapy increases local tumor control and leads to a prolonged progression free survival [4, 66].

Guidelines recommend 35.2 - 36 Gy to the craniospinal axis, divided into daily fractions of 1.6 or 1.8 Gy [19]. In addition to this, a boost to the posterior cranial fossa of up to 54 to 55.8 Gy should be performed [19].

Brandes et al. [67] performed a prospective trial with 26 adult patients who were divided into two groups depending on whether their tumor was classified as average or high risk. Average risk patients received radiotherapy without chemotherapy, whereas high risk patients were treated with radiochemotherapy after tumor resection. Radiotherapy was applied as craniospinal irradiation, with a total dose of 36 Gy divided into 20 fractions. Additionally, a boost to the posterior fossa of 18.8 Gy altogether, divided into 10 fractions, was applied. In the average risk group, 60% of the patients relapsed after a follow-up of almost 11 years. Overall survival in patients only treated with radiotherapy was 80% after 5 years [67]. In a small cohort of only 16 patients treated with radiotherapy, Buglione reported a 5 year overall survival of 75% and a 10 year survival of 67% [68].

Padovani et al. [69] presented a large retrospective series of adult medulloblastoma patients treated in several centers over a period of over 30 years. They reviewed the records of 253 adult patients. 66% had a classic histologic subtype and 30% had a desmoplastic tumor. After surgical tumor resection, 37% had a residual tumor [69]. Postoperative radiotherapy was performed in almost every patient with median doses of 35 Gy to the brain with a 54 Gy boost to the posterior fossa and also 35 Gy to the spinal axis [69]. A lower boost to the posterior fossa and a lower spinal radiation dose could be identified as prognostic markers for overall survival whereas a lower cranial radiation dose did not influence overall survival [69]. Also the duration of radiation did not influence the prognosis of the patients [69].

## Chemotherapy

Most published cohorts of adult medulloblastoma patients have been reviewed retrospectively and randomized trials are lacking. As children are usually treated with chemotherapy in addition to surgery and craniospinal irradiation, a great number of adult patients have also received chemotherapy.

For pediatric patients, Packer established polychemotherapy in the 1990s and the regimen consisted of vincristine, lomustine, and cisplatin. [18, 70, 71]

The role of chemotherapy is of great importance in adult patients and systemic therapy is recommended in current guidelines [19]. All adult patients with higher risk tumors should receive a systemic tumor treatment since survival rates are higher in patients with chemotherapy compared to patients only treated with radiotherapy [5, 72]. However, Brandes et al. recommend radiotherapy alone in average risk patients [73].

In the prospective multicenter NOA-07 trial, adult medulloblastoma patients were treated with radiochemotherapy followed by maintenance chemotherapy. [15] The treatment protocol consisted of radiotherapy with simultaneous application of vincristine. As a maintenance chemotherapy, vincristine, cisplatin, and lomustine were used. The aim of the study was to evaluate feasibility, efficacy, and toxicity of this regimen. Due to the rarity of medulloblastoma in adults, only thirty patients were enrolled on the study, and 67% of these had an SHH-activated tumor. Gross total resection was achieved in 50% of the patients and almost 75% had a complete response after radiochemotherapy [15]. Patients in this cohort underwent dose reduction due to treatment toxicity, including leukopenia, polyneuropathy, and ototoxicity. Progression free survival and overall survival at 3 years was 66.6% and 70%, respectively [15].

Friedrich et al. [74] observed adult patients with nonmetastatic medulloblastoma treated with radiochemotherapy and maintenance chemotherapy consisting of lomustine, vincristine, and cisplatin. During treatment, about 74% of the patients experienced neuropathy and 55% had hematotoxic adverse effects. Overall survival at four years was 89%. Negative prognostic factors were desmoplastic histology combined with a lateral tumor location and also the presence of residual tumor after surgery. The authors concluded that maintenance chemotherapy appears to improve overall survival, with an acceptable toxicity profile. Moreover, in this study, vincristine led to the most dose reductions [74].

Brandes et al. [67] conducted a study indicating radiochemotherapy for high-risk patients, as mentioned above. Risk stratification was applied, according to the Chang staging system and the presence of residual tumor. High-risk patients had T3b or T4 disease and/or a residual tumor after surgical resection [67]. The chemotherapy regimen was different compared to the two other trials outlined above. Firstly, the regimen consisted of mechlorethamine, vincristine, prednisolone, and procarbazine, and was later replaced by cisplatin, etoposide, and cyclophosphamide [67]. Moreover, high-risk patients received chemotherapy before and after craniospinal irradiation, whereas low-risk patients were only treated with radiotherapy [67]. Survival rates were inferior in patients with T3b or T4 disease [67].

Call et al. [38] retrospectively reviewed 66 adult patients over a median time of 6.7 years. Chemotherapy was applied to almost half of the cohort, and the regimen was mostly based on cyclophosphamide and/or cisplatin. They were unable to find a significant effect on overall survival. Patients with a classic histology had improved local tumor control when chemotherapy had been applied [38]. Interestingly, patients in this cohort were treated over decades, and those patients who underwent therapy in the 1970s did not have a statistically inferior outcome as compared to patients treated in the 2000s [38].

In a small cohort of only 11 adult patients with high risk tumors, the use of pre-radiation chemotherapy was studied [75]. High-risk was defined as residual tumor greater than 1cm^2^ or subarachnoid dissemination. Chemotherapy consisted of cisplatin, cyclophosphamide, etoposide, and vincristine. Response rates before radiation were low, and overall survival did not exceed 60% [75]. The authors of this trial did not see a clear benefit of chemotherapy before radiation in their series with a limited number of patients. Table 2 summarizes selected trials dealing with adult medulloblastoma.

**Table 2:** Overview over selected clinical trials concerning adult medulloblastoma.

**Overview of selected adult medulloblastoma cohorts**				
**Author**	**Year**	**No. of patients**	**Special characteristics**	**Overall survival**
Brandes et al.	2007	26	Adjuvant Chemotherapy (CRT) vs. Radiotherapy (RT)	80% (CRT)/73%(RT)/5 yrs
Padovani et al.	2007	253		75%/5 yrs - 55%/10 yrs
Friedrich et al. (HIT-2000)	2013	70	Adjuvant Chemotherapy	89%/4 yrs
Call et al.	2014	66		74%/5 yrs
von Bueren et al. (HIT-2000)	2015	23	Metastatic disease	91%/4 yrs
Kann et al. (NOA-07)	2017	751	Adjuvant Chemotherapy (CRT) vs. Radiotherapy (RT)	86.1% (CRT)/71.6%(RT)/5 yrs
Moots et al.	2018	11	Pre-radiation Chemotherapy	55%/5 yrs

The studies described above used a combination of several chemotherapeutic agents. Also temozolomide, which is widely used in the treatment of high grade gliomas, [76] has been subject of several medulloblastoma trials, mostly in childhood patients. Temozolomide was used either as a monotherapy or combined with other antiproliferative substances, e.g. irinotecan or bevacizumab, [77–80]. Investigations have also focused on response rates for pediatric patients with disseminated or progressive disease, and response rates of more than 40% have been reported [81].

SHH-activated medulloblastomas can be treated with vismodegib as a targeted therapeutic agent, [82, 83] and the drug is available orally [82]. *In vitro* analyses have demonstrated a strong antiproliferative effect of vismodegib alone and vismodegib in combination with PI3K/AKT/mTOR-inhibition and/or cisplatin [84]. The *in vitro* studies indicate a chemosensitizing function of vismodegib, which increases the cytotoxic impact of cisplatin *in vitro* [84].

Vismodegib leads to an increased progression-free and increased overall survival [83, 85, 86]. Initially developed to treat basal cell carcinoma, vismodegib has been approved as a chemotherapeutic agent for recurrent SHH-medulloblastoma resulting in better response rates in almost half of the patients [86]. Rates of adverse events are reported to be low, largely confined to nausea and a reduction of white blood cell count [86]. Case reports have shown impressive responses to vismodegib in occasional patients [85]. Adverse effects include muscle spasms, nausea, dysgeusia, weight loss, and fatigue [83].

## Disseminated disease

Medulloblastoma is a tumor entity that tends to seed metastases in the spinal axis. The cerebrospinal fluid should be investigated for tumor dissemination in the initial staging, and this is also recommended during follow-up exams. Moreover, extraneural metastases have been reported in parts of patient cohorts and case reports [87–90]. Metastatic spread is more common in infants and children [23]. Extraneural metastases also occur in adult medulloblastoma patients and a few case reports have been published [87, 88, 91]. Primary tumors and metastatic lesions are usually present with the same molecular subgroups, as analyzed by gene expression, methylation analysis, and immunohistochemistry [92]. The most common sites for metastatic spread outside the CNS are the bone and bone marrow [90].

The HIT 2000 trial aimed to achieve an appropriate chemotherapy treatment protocol for adult patients with metastatic or disseminated disease [93]. Patients received either postoperative chemotherapy followed by radiotherapy and maintenance chemotherapy, or maintenance chemotherapy alone [93]. During the four years of follow-up, the event free survival was about 52%, with an overall survival of over 90% [93]. In this small cohort of slightly more than 20 patients, patients with an anaplastic subtype did not survive, whereas patients with classic or desmoplastic histology had a more favorable overall survival [93]. Treatment toxicities were mainly neurotoxicity, occurring in half of the patients. Intrathecal application of methotrexate was reported without adverse effects in most patients [93].

## Surveillance, prognostic factors, and survival

Tumor relapse can occur months and years after primary therapy [94]. For this reason, systematic surveillance and follow-up examinations are of great importance to achieve the best long-term prognosis for medulloblastoma patients after the first tumor treatment. The aim of follow-up is to check for local relapse or tumor progression, metastatic spread, and treatment toxicity, and side effects should be regarded as well. Follow-up examinations should be carried out every three months during the first 5 years, including cerebral and spinal imaging as well as cerebrospinal fluid examination [19]. It is sensible to perform follow-up examinations until 10 years after diagnosis [19].

Brandes et al. [67] found a high risk for recurrent disease, even after 7-10 years after completed tumor treatment. In the SEER database, the survival of adult medulloblastoma patients was 79.9% at 2 years, 64.9% at 5 years and 52.1% at 10 years [4]. Other investigations have revealed a number of factors influencing survival for adult medulloblastoma. These include the male sex, molecular subtype of the tumor, Chang risk stratification, presence or absence of metastases, extent of resection, irradiation dose, maintenance chemotherapy, and Karnofsky performance status [12, 72, 95]. WNT-positive tumors appear to have an excellent prognosis among all age groups [23]. SHH-tumors only show superior survival if the histological subtype is desmoplastic [23].

Giordana et al. [96] could not find a correlation between survival and the presence of anaplasia in their series of adult medulloblastoma samples. Another meta-analysis could not find a correlation between histological subtype and overall survival [5].

Kann et al [97]. demonstrated an improved survival for adult patients treated with craniospinal irradiation plus chemotherapy as compared to patients only receiving radiotherapy. They also found an M0-stage to be a positive prognostic factor. An irradiation dose of 36 Gy was associated with a favorable outcome. Poly- versus mono-agent chemotherapy did not influence prognosis in this trial [97]. Other data support a prognostic improvement through chemotherapy at first line [5].

Padovani et al. [69] found metastatic disease, tumor invasion to brainstem and fourth ventricle, and also reduced radiation dose to the posterior fossa, as negative prognostic factors. They also reported an overall survival of 55% after 10 years. Other investigators also found metastatic disease at tumor recurrence negatively influenced survival, whereas the presence of metastases at the time of initial diagnosis has not been shown to have a negative influence [5].

## CONCLUSIONS

Adult medulloblastoma patients require a multimodal treatment to achieve the best prognostic results. An interdisciplinary team of neurosurgeons, neuroradiologists, anesthesiologists, neuropathologists, radiotherapists, and oncologists is needed to deal with this complex tumor entity.

Adult medulloblastoma is rare, and trials with large numbers of patients are lacking, especially randomized controlled trials. Nevertheless, research dealing with childhood medulloblastoma has delivered a great amount of knowledge concerning tumor biology, points of application for treatment strategies, and prognostic factors. However, there is evidence that there are differences between childhood and adult medulloblastoma, both in terms of their molecular pathology and treatment. Prospective research for molecular pathological characteristics of this tumor will deliver opportunities for more complex and patient-orientated treatment strategies, including targeted therapeutics depending on the molecular tumor biology of individual patients.

In the future, further research is needed to increase surgical success and safety for cerebellar mass lesions, such as intraoperative imaging and fluorescence, functional imaging, and neuromonitoring. Neurosurgical tumor resection will still be relevant in treatment protocols for medulloblastomas to reduce complications and concerns caused by the tumor mass.
